# First documentation on herpetofauna diversity in Gunung Belumut Amenity Forest, Peninsular Malaysia: Implications for conservation in an Environmentally Sensitive Area (ESA)

**DOI:** 10.3897/BDJ.12.e108476

**Published:** 2024-01-09

**Authors:** Farah Farhana Ramli, Kaviarasu Munian, Nur Aina Amira Mahyudin, Nursyuhada Othman, Hidayah Haris, Nurfatiha Akmal Fawwazah Abdullah-Fauzi, Nur Hartini Sariyati, Mohd Lokman Ilham-Norhakim, Muhammad Abu Bakar Abdul-Latiff

**Affiliations:** 1 Environmental Management and Conservation Research Unit (eNCORe), Faculty of Applied Sciences and Technology, Universiti Tun Hussein Onn Malaysia (Pagoh Campus), 84600, Pagoh, Johor, Malaysia Environmental Management and Conservation Research Unit (eNCORe), Faculty of Applied Sciences and Technology, Universiti Tun Hussein Onn Malaysia (Pagoh Campus), 84600 Pagoh, Johor Malaysia; 2 Zoology Branch, Forest Biodiversity Division, Forest Research Institute Malaysia (FRIM), 52109, Kepong, Selangor, Malaysia Zoology Branch, Forest Biodiversity Division, Forest Research Institute Malaysia (FRIM), 52109 Kepong, Selangor Malaysia; 3 Kim Ichthyologist Centre, Kg Parit Samsu, Jalan Temenggong Ahmad, 84150, Parit Jawa, Muar, Johor, Malaysia Kim Ichthyologist Centre, Kg Parit Samsu, Jalan Temenggong Ahmad, 84150, Parit Jawa Muar, Johor Malaysia; 4 Akim Fishes Enterprise, 81P Pesta 2, Kg Kenangan Tun Dr Ismail, 84000, Muar, Johor, Malaysia Akim Fishes Enterprise, 81P Pesta 2, Kg Kenangan Tun Dr Ismail, 84000 Muar, Johor Malaysia

**Keywords:** Amphibia, reptile, protected area, species richness, biodiversity

## Abstract

Malaysia is blessed with lush tropical rainforests that harbour an exceptional diversity of amphibians and reptiles. However, compared to other animal groups, amphibians and reptiles have received limited attention in research, despite their ecological significance. With amphibians and reptile species having been declining rapidly due to anthropogenic activities, there is a pressing need to conserve these species and their habitats. Environmentally Sensitive Areas (ESAs) are designated regions that are beneficial due to their critical role in providing essential ecosystem services and serving as repositories of biodiversity. Nonetheless, the classification of ESAs in Malaysia lacks biological elements and only focuses on physical attributes. To enhance the current ESA classification framework by integrating biological components, there is an urgent need to obtain information on diversity and habitat in Malaysia. Therefore, the objectives of this study were twofold: to determine the diversity of amphibians and reptiles in Gunung Belumut Amenity Forest and to conduct a comparative analysis between the herpetofauna assemblages in Gunung Belumut with other forest reserves in Peninsular Malaysia. The survey was carried out between March and June 2022, with additional sampling conducted in February 2023. The Visual Encounter Survey (VES) and pitfall trap methods were employed to survey the herpetofauna species, focusing on both aquatic and terrestrial habitats within the study area. A total of 210 individuals representing 38 species of herpetofauna were recorded, comprising 18 amphibian and 20 reptile species. Amongst the observed species, *Limnonectesblythii* was the most frequently encountered amphibian, with 59 individuals observed, while the dominant reptile species was *Cyrtodactylusconsobrinus*, represented by eight individuals. This pioneering study serves as a vital baseline documentation of the amphibian and reptile assemblages in Gunung Belumut Amenity Forest. It provides valuable information for identifying extant herpetofauna species, including those of potential conservation concern or rarity. These findings contribute to ongoing conservation efforts dedicated to the preservation of herpetofauna within the region. By understanding the diversity and distribution patterns of amphibians and reptiles in Gunung Belumut, effective conservation strategies can be developed to protect these species and their habitats.

## Introduction

The tropical rainforest of Malaysia is one of the most complex and diverse forests in the world, not only in terms of species diversity, but also in terms of habitat and ecosystem diversity. Located nearest to the Equator, Malaysia is blessed with optimum sunlight and precipitation levels that can support its tropical rainforest (Zakaria et al. 2022). These warm and humid conditions provide an excellent environment for animals to flourish resulting in high species diversity. As one of the most biologically diverse nations in the world, the tropical forests in Malaysia are home to large populations of year-round active herpetofauna species (Nazir-Khan and Mohd 2007). Herpetofauna is a group of animals that includes amphibians and reptile species that play an effective role as a biological indicator and component in an ecosystem (Hammond et al. 2016). To date, there are approximately 242 species of amphibians and 567 species of reptiles documented throughout Malaysia including Peninsular Malaysia, Sabah and Sarawak (Convention Biological Diversity 2019). Between 2000 and 2021, findings on new species of herpetofauna within Malaysia have been rising, with at least 70 species being newly discovered (Chan et al. 2014, Matsui 2019, Quah et al. 2020, Chan et al. 2022, Grismer et al. 2022, Zijia et al. 2023). Such a scenario indicates that the diversity of herpetofauna in Malaysia is still far from completion and emphasises the imperative future research on its biodiversity within the region.

Herpetofauna is an effective biological indicator that plays a vital role in the environment (Hammond et al. 2016). They also significantly contribute to the preservation of biodiversity at the intermediate level of the ecosystem food chain, acting as both predators of terrestrial and aquatic insects, as well as prey for fish, birds and mammals (Valencia et al. 2013, Cortés-Gomez AM et al. 2015). Furthermore, because of their short migration distance and limited ability to disperse due to their limited range of motion in comparison to other vertebrates, herpetofauna species are known to be vulnerable to habitat destruction and climate change (Hoffman and Sgro 2011).

According to Omran and Schwarz-Herion (2020), Malaysia has some of the highest deforestation rates in the world with logging concessions covering most of the country’s remaining forest. A previous study conducted by Izam et al. (2019) showed that amphibian species richness and abundance began to decline, particularly when the logging activities were carried out to clear the forested area to make way for a reservoir area. Moreover, as a consequence of the rapid economic growth witnessed in recent decades, the forest cover in Peninsular Malaysia has experienced a significant decline. From its peak of 80% in 1940, the forest cover diminished to 60% in 1971 and, by the end of 2019, only 54.9% of the total land area of Malaysia was under forest cover (Aiken 1994, Ministry of Natural Resources, Environment, and Climate Change 2022). To protect its natural and cultural resources, Malaysia has employed an approach similar to the land-use-based strategies adopted globally, known as Environmentally Sensitive Areas (ESA).

ESA is an area that needs specialised management to protect habitats, wildlife, ecosystems, natural processes and scenic landscapes, as well as historical and cultural interests (Chen and O'Yang 2006). The implementation of ESAs is crucial for achieving a balance between biodiversity conservation and land-use planning, guided by the principles of sustainable development. However, the current classification of ESAs in Malaysia primarily focuses on physical attributes, such as slope gradients, elevations and risk levels, thereby potentially neglecting the biological aspects of the environment (Shahfiz et al. 2021). This evidence highlights the need for improvements to effectively fulfil the intended objectives of ESA classification. Enhancing the inclusion of biological and ecological components within the ESA classification requires the availability of essential data about species diversity, abundance, distribution patterns, species composition or assemblages and threat statuses (Munian et al. 2023). Unfortunately, such comprehensive biological information in Malaysia remains insufficient and it is crucial to acknowledge the pressing need for continuous documentation across various dimensions of the biological components. Therefore, the assessment of the current status of biological components including the herpetofauna diversity and also the level of information available, especially in Peninsular Malaysia, is vital in developing effective ESA classification to protect species and their habitats.

In order to initiate the inclusion of biological components into ESA, this study is aimed to document the diversity of the herpetofauna within the unexplored terrain of Gunung Belumut Amenity Forest in Johor, making it the first documentation of herpetofauna diversity in this specific locale. Besides that, we intended to compare the herpetofauna assemblage found in Gunung Belumut with other forest reserves representing different ecoregions in the east and west coasts (northern region and southern region) of Peninsular Malaysia. By acquiring valuable data through this study, relevant authorities and stakeholders can be equipped with essential information to come up with appropriate measures aimed at enhancing the habitat conditions necessary for conserving the diverse array of herpetofauna species in Gunung Belumut Amenity Forest and other similar habitats.

## Material and methods

### Study Area

**Figure 1.**
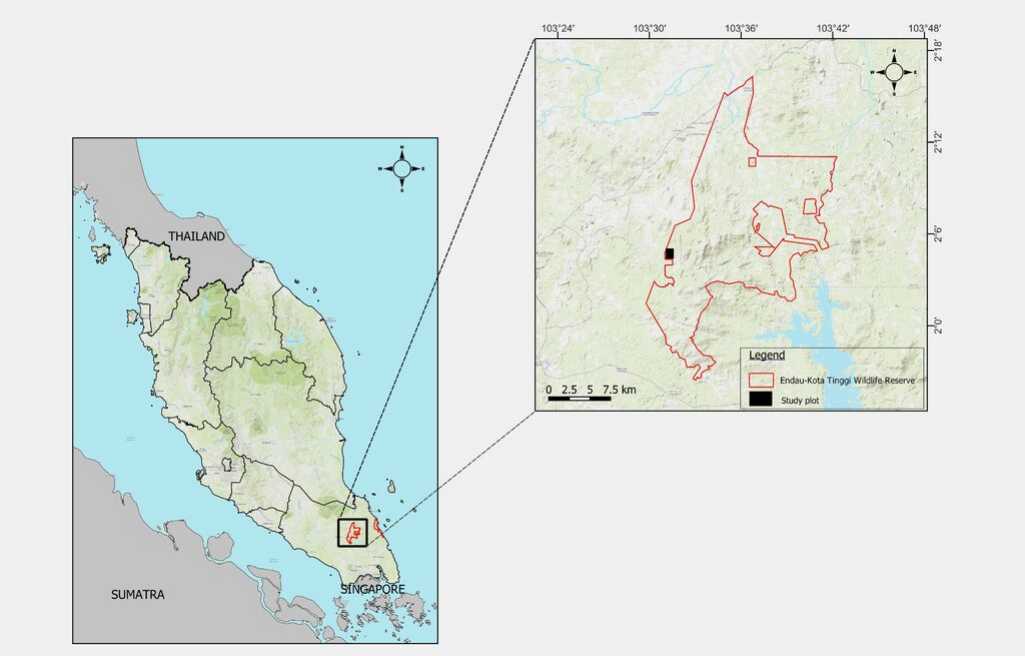
The location of Gunung Belumut Forest Amenity, Peninsular Malaysia. The black square indicates the study plot.

Gunung Belumut situated at coordinates N 2°3ʹ56.016ʺ, E 103°31ʹ41.138” (Fig. 1). Gunung Belumut stands as an isolated mountain, characterised by an elevation of 1010 m above sea level and having the third highest peak in Johor (Fig. 1). Positioned approximately 30 km away from the closest town, Kluang, this mountain is encompassed within Kluang Forest Reserve, specifically representing a hill dipterocarp forest. The surrounding area of Gunung Belumut comprises the Felda Ulu Dengar region, which is primarily characterised by expansive oil palm plantations. Over the years, various studies focusing on biodiversity have been carried out within the realm of Gunung Belumut, encompassing studies of the diversity of beetles (Abdullah et al. 2012), freshwater fish (Chow et al. 2016), macroinvertebrates (Eh-Rak et al. 2010, Zakaria 2019) and butterflies (Rahman and Mohamed 2020).

### Herpetofauna Sampling and Collection

The survey was carried out on six separate occasions spanning from March to June 2022 with additional sampling in February 2023. The survey focused on sampling along the stream and forest trails, using a combination of drift-fenced pitfall traps and opportunistic searches as the primary methodologies (Enge. 2001, Fisher et al. 2008, Rahman et al. 2022). In order to capture terrestrial confined herpetofauna, we deployed "Y" shaped drift-fenced pitfall traps comprised of a plastic bucket sized 18 litres. We set out a total of three replicates, with nine plastic buckets for each replicate, buried securely into the ground. All the replicates were located in a 400 m x 200 m plot, where the collection of other taxa of vertebrates including small mammals and birds was carried out concurrently (Fig. 1). Each of the buckets was punctured at the bottom to allow proper drainage and subsequently buried flush with the ground surface. These traps were left open for consecutive periods of five days, with daily inspections. The visual encounter survey (VES) procedure entailed active searching of herpetofauna using wide-beam headlights to enhance visibility during night-time observations. This method involved systematically traversing restricted areas along the designated trails at a specific time at night. Additional techniques, such as the utilisation of sweep nets and snake tongs, facilitated the active search for herpetofauna across forest trails, arboreal habitats, beneath rock formations and within riverine environments or bodies of water. Each of the captured individuals was carefully placed in a separate plastic bag prior to examination.

After specimen capture, the animals were carefully brought back for measurement, identification and photographed. Morphological measurements were taken, encompassing snout-vent length (SVL) and total length (TL) using a caliper and all the data were recorded systematically. Identification of the specimens was done by referring to Berry (1975), Tweedie (1983), Lim and Das (1999), Chan et al. (2010a), Grismer (2008), Grismer (2011a), Grismer (2011b), and Das (2015). After completion of specimen examination, the majority of the specimens were released back into their original habitat. However, a few selected individuals representing each species were retained as voucher specimens. The collection of voucher specimens was particularly important in facilitating the identification of unknown taxa and obtaining tissue samples for taxonomic groups for further taxonomic classification. The taxonomic classification and nomenclature follows Frost (2023). A wildlife research permit was obtained from the Department of Wildlife and National Park, Peninsular Malaysia (PERHILITAN, Research Permit No: B-00298-15-22 & B-00381-15-22) to allow us to conduct a survey and voucher specimen collection.

A comprehensive compilation of the recorded herpetofauna species was generated, enabling a comparative analysis with similar studies conducted in other amenity forests and forest reserves. The available literature encompassed various locations, including Bukit Perangin Forest Reserve (BPFR) (Ibrahim et al. 2012), Bukit Panchor State Park (BPSP) (Nur-Hafizah 2011, Quah et al. 2013), Batu Hampar Recreational Forest (BHRF) (Zijia et al. 2021), Belum-Temenggor Forest Reserve (BTFR) (Kiew et al. 1995, Diong et al. 1995, Lim et al. 1995a, Lim et al. 1995b, Norsham et al. 2000, Sukumaran 2002, Grismer et al. 2004, Hurzaid et al. 2013), Sg. Deka Tembat Forest Reserve (SDTFR) (Mohd-Izam et al. 2021), Gunung Senyum Forest Reserve (GSFR) (Davis et al. 2018), Pasoh Forest Reserve (PFR) (Kiew et al. 1996, Lim and Norsham 2003, Norsham and Sukumaran 2006, Chan et al. 2009) and Gunung Panti Forest Reserve (GPFR) (Leong 2004, Yong 2006, Chan et al. 2010b).

### Data Analysis

We constructed a species accumulation curve for herpetofauna species in the study site in order to determine the completeness of the sampling efficiency. Our decision to opt for an individual-based approach, rather than a sample-based approach, was driven by our main objective of estimating and comparing species richness (the total species count at a specific location), as opposed to species density (the number of species per unit area) (Colwell et al. 2012). The curve was constructed using the Hill number approach (q = 0) iNEXT package by Hsieh et al. (2016).

We calculated and compared species diversity for herpetofauna using several indices, such as species richness, Shannon-Wienner Index, Evenness Index and Dominance Index. Additionally, we estimated the species richness of herpetofauna in GBAF, based on the Chao 1 estimator. Lastly, to compare the assemblages of herpetofauna in GBAF with selected amenity forests and forest reserves in Peninsular Malaysia, the Bray-Curtis Similarity Index was calculated to show the similarity in the composition of amphibians and reptiles. All the analyses were done using the vegan package (Oksanen et al. 2021) in R Studio (RStudio Team 2021).

## Results

A total of 210 individuals were documented during the study, comprising 172 individuals of amphibians and 38 individuals of reptiles, representing 18 distinct species of amphibians and 20 species of reptiles (Table 1). The relative abundances of amphibian species exhibited a range of 0.6% to 34.3%, with *Limnonectesblythii* emerging as the dominant species, accounting for a total of 59 recorded individuals. In the case of reptiles, relative abundances varied between 0.5% and 3.8%, with *Cyrtodactylusconsobrinus* being the most dominant species, comprising a total of eight documented individuals.

Table 2 presents the calculated diversity indices for both amphibians and reptiles. In terms of amphibian species diversity, the Shannon Diversity Index recorded a value of 2.15, further supporting the presence of a diverse amphibian community. The Dominance Index was low, measuring 0.18, indicating a balanced distribution amongst species. Additionally, the species Evenness Index yielded a value of 0.48, further supporting the equitable distribution of amphibian species within the study area. The Chao 1 estimator recorded a value of 21.33.

For reptiles, the Shannon Diversity Index yielded a value of 2.67, further supporting the presence of a diverse reptile assemblage. Similar to amphibians, the Dominance Index for reptiles was low at 0.10, indicating a balanced distribution of species. The species Evenness Index for reptiles recorded a value of 0.72, implying an equitable distribution of reptile species within the study area. The Chao 1 estimator recorded a value of 42.75.

**Figure 2.**
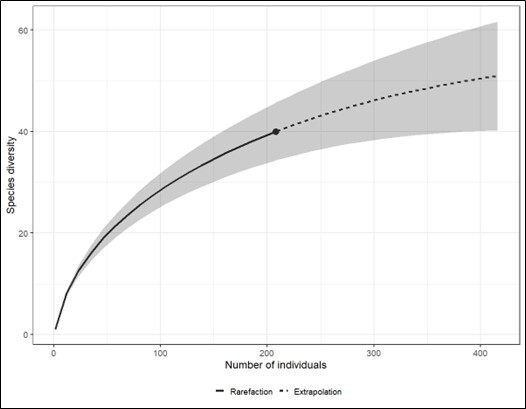
Herpetofauna species sample-based rarefaction curve at Gunung Belumut Amenity Forest, Johor.

Fig. 2 depicts the species accumulation curve, based on individual samples of herpetofauna collected from GBAF. The observed pattern in the curve suggests that the documented diversity of herpetofauna has not yet reached its asymptote, indicating that the conducted surveys have been insufficient. This result is further supported by the Chao I estimator, which indicates that the expected species richness for amphibians is almost 92% complete, while for reptiles, the study has only managed to document approximately 46% of the expected species richness.

**Figure 3.**
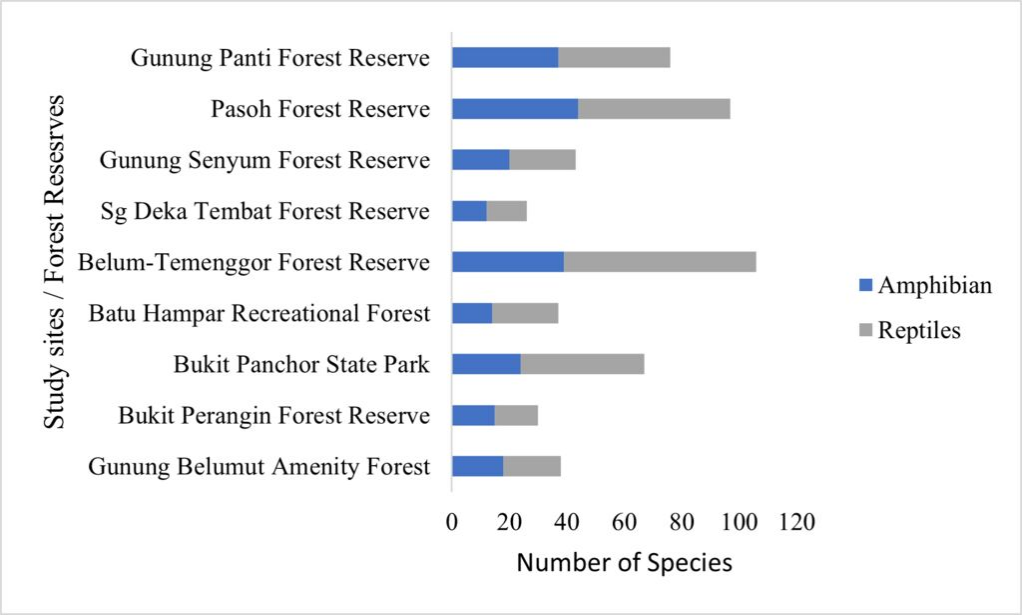
Number of herpetofauna species recorded in GBAF, BPFR, BPSP, BHRF, BTFR, SDTFR, GSFR, PFR and GPFR.

**Figure 4.**
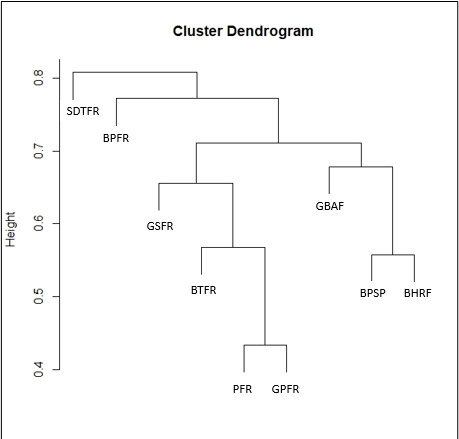
Cluster dendrogram for herpetofauna species assemblages in Gunung Belumut Amenity Forest and other related forest reserves in Peninsular Malaysia.

**Figure 5.**
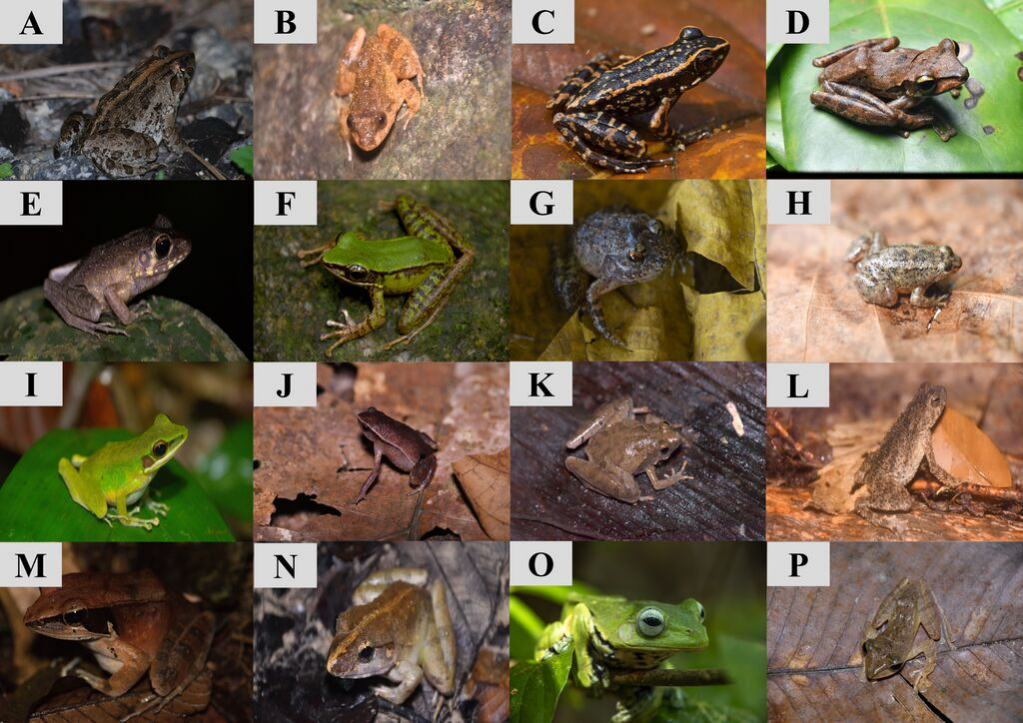
Collection of amphibian species that were recorded in GBAF. A: *Fejervaryalimnocharis*; B: *Limnonectesdeinodon*; C: *Hylaranasundabarat*; D: *Polypedatesmacrotis*; E: *Hylaranabaramica*; F: *Odorranahosii*; G: *Limnonectesplicatellus*; H: *Occidozygamartensii*; I: *Hylaranalabialis*; J: *Kalophrynuslimbooliati*; K: *Microhylabutleri*; L: *Phrynoidisasper*; M: *Hylaranamiopus*; N: *Limnonectesblythii*; O: *Rhacophorusnorhayatiae*; P: *Polypedatesleucomystax*.

**Figure 6.**
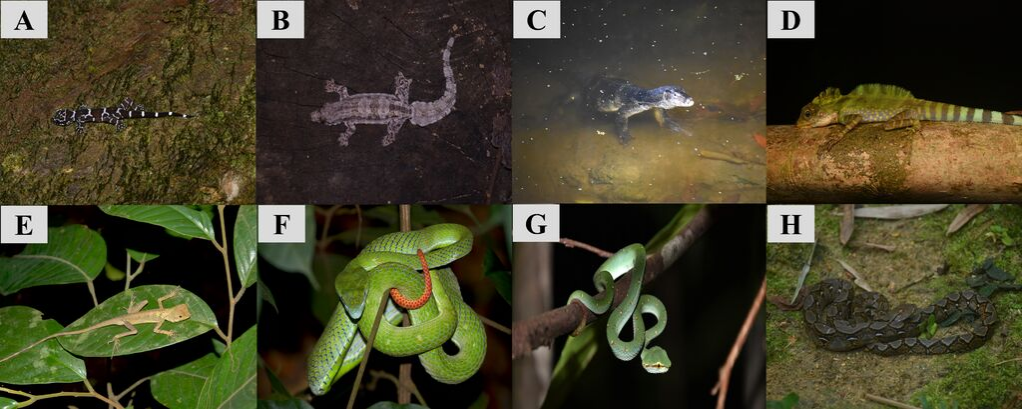
Collection of reptile species recorded in GBAF. A: *Cyrtodactylusconsobrinus*; B: *Hemidactyluscraspedotus*; C: *Varanussalvator*; D: *Gonocephalusgrandis*; E: *Aphaniotisfusca*; F: *Trimeresurushageni*; G: *Tropidolaemuswagleri*; H: *Malayopythonreticulatus*.

Furthermore, to assess the diversity of herpetofauna in GBAF, comparative and similarity analyses were conducted with other forest reserves in Peninsular Malaysia (Suppl. material 1). By referring to published checklists of the herpetofauna from various forest reserves, the BTFR exhibits the highest species richness, documenting a total of 106 species. Then, PFR recorded 97 species of herpetofauna, while GPFR documented 76 species. Notably, BPSP accounted for 67 recorded species, GSFR reported 43 species and BPFR documented 30 species. Similarly, BHRF recorded 37 species and SDTFR documented 26 species (Fig. 3). A cluster dendogram, based on the Bray-Curtis Similarity Index (Fig. 4), indicated that herpetofauna assemblages in GBAF were almost similar to BHRF in the State of Kedah and BPSP in the State of Penang and clustered together with GSFR, BTFR, PFR and GPFR. In addition, Fig. 5 shows the voucher photos for amphibians and Fig. 6 shows the voucher photos for reptiles.

## Discussion

### Herpetofauna diversity at Gunung Belumut Amenity Forest

The family Dicroglossidae shows the highest abundance of amphibian species in this study, with *L.blythii* being the most frequently recorded species (51 individuals). The distribution of *L.blythii* extends across Southeast Asia, including Vietnam, Thailand, Peninsular Malaysia and Sumatra and this species is commonly found within an elevation range from sea level to 1200 m above sea level (IUCN 2015). In Peninsular Malaysia, *L.blythii* is typically encountered along rivers and streams within primary and secondary forests (Berry 1975, Ibrahim et al. 2008b). It is also often observed on the forest floor, away from water sources (Grismer 2011a). Previous studies by Chan et al. (2010b), Norhayati et al. (2011) and Chan et al. (2019) have reported a higher distribution of this species in lowland and hill dipterocarp forests. In addition, it is worth noting that certain species found in this habitat are human-commensal species. According to Inger (2005), the presence of these species is typically associated with disturbed habitat. The commensal species recorded in GBAF include *Fejervaryalimnocharis*, *Microhylabutleri* and *Polypedatesleucomystax* (Shahriza and Ibrahim 2014a). The presence of these species suggests that the habitat in GBAF has experienced slight disturbances, which is evident from the presence of oil palm plantations and other agricultural activities in the surrounding areas of GBAF.

The herpetofauna inventory also recorded various forest frog species, such as *Odorranahosii*, *Phrynoidisasper* and several other species that are known to inhibit GBAF (Shahriza and Ibrahim 2014a). *O.hosii* and *P.asper* are species associated with clean water environments, particularly swift, clear rocky streams, which are one of the habitat types found within GBAF (Dijk et al. 2004, Ibrahim et al. 2012). During sampling, most of the individuals were observed perched on rocks, vegetation and the grounds along the trail and the stream. Amongst the notable findings in GBAF was the presence of *Rhacophorusnorhayatiae*, a rare species of tree frog widely distributed in Peninsular Malaysia. *R.norhayatiae* primarily inhabits lowland and hill forests, residing on trees up to 7 m above the forest floor, often near temporary water bodies, such as pools and puddles and occasionally in water-filled tyre tracks on logging roads (Chan and Grismer 2010). While *R.norhayatiae* is typically found at elevations up to 550 m above sea level, individuals have been observed as high as 1500 m above sea level in Tanah Rata, Cameron Highland, Pahang (Berry 1975). Its range extends beyond Peninsular Malaysia to the south and a small area of west-central Thailand, near the Myanmar border (Dring 1979, Chan-ard 2003).

For reptiles, this study documented a total of 20 species, with *C.consobrinus* being the most commonly observed reptile species. *C.consobrinus*, known as a forest gecko, is frequently encountered in lowland dipterocarp forests, characterised by mature trees (Anandarao 2011), which closely resemble the forest composition of Gunung Belumut. This species has also been recorded in other mountainous regions of Peninsular Malaysia including Gunung Panti (Chan et al. 2010b), Gunung Inas (Shahrudin et al. 2013), Gunung Tebu (Sumarli et al. 2015) and Gunung Korbu (Chan et al. 2019). Furthermore, five reptile species were recorded with only one individual each during the sampling activities. These species include *Gonyosomaoxycephalum*, *Dendrelaphiscyanochloris*, *Subdolusepsbowringii*, *Trimesaurushageni* and *Tropidolaemuswagleri*. The limited occurrence of these species in the study area may be attributed to their elusive and secretive behaviour, for which the use of appropriate sampling techniques is crucial for detection. For instance, *G.oxycephalum*, *D.cyanochloris* and *T.hageni* are often spotted hanging from trees (Sumarli et al. 2015, Zijia et al. 2021) and *T.wagleri* is commonly found in bamboo stalks (Grismer 2011a, Sumarli et al. 2015). Such behaviour and microhabitats can pose challenges during sampling sessions, potentially resulting in the under-representation of these species. Furthermore, the presence of various abiotic factors, including microclimate and microhabitat within the GBAF, influenced the diversity of reptile families. Many lizard species, for instance, exhibit a preference for occupying forested areas, where they often perch on tree trunks and navigate rock surfaces (Ibrahim et al. 2012) and certain species, such as turtles rely heavily on permanent water sources like streams and ponds (Ibrahim et al. 2012). These specific microhabitat requirements contribute to the observed variations in reptile family composition within GBAF.

### Comparison of Herpetofauna assemblage

BTFR stands out with the highest reported species richness of herpetofauna, totalling 105 species. This notable richness could be attributed to the comprehensive and extensive sampling efforts carried out at BTFR over five years (1995, 2000, 2002, 2004 and 2012) (Kiew et al. 1995, Diong et al. 1995, Lim et al. 1995a, Lim et al. 1995b, Norsham et al. 2000, Sukumaran 2002, Grismer et al. 2004, Hurzaid et al. 2013). In contrast, the limited sampling period and frequency at GBAF resulted in a low number of species. Only five sampling trips were conducted within three months period at GBAF compared to the long sampling durations implemented in other study sites. The constrained timeframe made it challenging to cover sufficiently large sampling areas, thereby reducing the chances of encountering a greater number of species (Ibrahim et al. 2012). Additionally, the relatively fewer microhabitats present within GBAF compared to other study sites could potentially contribute to the low number of species richness along with the limited accessibility of certain areas within GBAF which may have restricted the opportunity for species encounters compared to more expansive areas in other locations. For instance, BPSP (Nur-Hafizah 2011, Quah et al. 2013) encompasses a diverse range of habitats, including forests, streams, swamps and granite boulder caves which can support a greater assemblage of species compared to GBAF, primarily composed of forests and streams.

Notably, *Leptobrachiumhendricksoni*, a member of the Megophyridae family, was observed at all sampling sites, except for GBAF and GSFR, despite both sites sharing similar habitat characteristics. Additionally, certain gecko species, *Cyrtodactylusquadrivirgatus* and *Gehyramutilata*, were found in other study locations but were absent from GBAF. According to Egan et al. (2016), they predicted that nocturnal geckos would employ bands as a form of camouflage, as bands are expected to offer a particularly effective means of blending with the surrounding background in common sheltering environments, such as rocky crevices and leaf litter and these environments exhibit diverse depth profiles, resulting in pronounced shadows that generate stark contrasts. This might contribute to a low number of gecko species in GBAF. Moreover, several diurnal and arboreal snake species, such as *Xenochrophistrianguligerus*, were encountered at most sampling sites, excluding GBAF. Snakes have been known to exhibit a few types of reverse crypsis to hide their presence from potential predators and prey (Greene 1988, Greene 1997). Moreover, the colouration pattern exhibited by certain species enables effective camouflage, further reducing their visibility (Ibrahim et al. 2013, Shahriza and Ibrahim 2014b). Nonetheless, it is worth noting that GBAF recorded some species exclusively, which were not encountered in the other study sites. These unique findings include *Polypedatesdiscantus* and *R.norhayatiae* amongst the amphibians and *Cyrtodactylussworderi*, *D.cyanochloris* and *Ptyasfusca* amongst the reptiles.

The decline of amphibians and reptiles on a global scale can be attributed to a range of factors, including physical habitat destruction, direct human alterations, exposure to ultraviolet radiation, acidification, chemical pollutants, diseases and climate and weather changes (Gardner 2001). However, despite the significance of this issue, there remains a lack of information regarding the herpetofauna species, particularly in the context of Peninsular Malaysia. Sufficient knowledge about species is essential for effectively addressing conservation challenges. Consequently, there is an urgent need to assess the present status of herpetofauna diversity and the available information pertaining to amphibian and reptile species in Peninsular Malaysia. Population monitoring and biodiversity studies often face limitations in terms of resource availability and the extent of effort that can be invested (Gardner et al. 2007). Consequently, it is crucial to employ sampling techniques that maximise the effectiveness of these studies in achieving their objectives (Ribeiro-Júnior et al. 2008). However, several factors, including geographical location, sampled habitats, target taxa, study aims and duration, can influence the efficacy of sampling approaches (Doan 2003). According to Ryan et al. (2002), no universally applicable sampling method can capture the entirety of herpetofauna species within a community, but certain approaches may provide more accurate estimations of abundance or diversity compared to others. In addition, traditional methods of sampling and identification, based on direct observation or voucher specimens, are now increasingly being complemented by DNA-based techniques. Considering the vulnerability of herpetofauna species to various threats and their relatively limited comprehensive study compared to other vertebrate groups, it is imperative to establish long-term monitoring programmes and gather baseline data. This will ensure the continuity of these species within the environmentally sensitive areas of Peninsular Malaysia.

## Conclusions

The surveys in GBAF have yielded valuable insights into the herpetofauna diversity of the area, resulting in the documentation of 42 species. The compilation of checklists not only serves as an interesting finding, but also increases the exploration for rare or infrequently encountered species, providing valuable information on species' presence, abundance and distribution. Such comprehensive surveys hold the potential for discovering a greater number of previously unknown records. Preserving GBAF in its undisturbed state is of paramount importance due to its significant role as a habitat for a diverse array of herpetofauna species. Ensuring that any future development in the recreational area does not compromise or degrade the pristine ecosystem is crucial to the continued survival and existence of these herpetofauna populations. Given their recognised contributions to the ecological processes within the tropical forest ecosystem, safeguarding the integrity of this undisturbed habitat remains imperative.

## Supplementary Material

003F4F57-7D23-5766-AB18-C3D5B5D57577 10.3897/BDJ.12.e108476.suppl1 Supplementary material 1 Comparison of herpetofauna diversity Data type Checklist of herpetofauna assemblage Brief description The assemblage of herpetofauna recorded in GBAF, BPFR, BPSP, BHRF, BTFR, SDTFR, GSFR, PFR and GPFR. Comparison of herpetofauna diversity in Gunung Belumut Amenity Forest (GBAF), Bukit Perangin Forest Reserve (BPFR), Bukit Panchor State Park (BPSP), Batu Hampar Recreational Forest (BHRF), Belum-Temenggor Forest Reserve (BTFR), Sg, Deka Tembat Forest Reserve (SDTFR), Gunung Senyum Forest Reserve (GSFR), Pasoh Forest Reserve (PFR) and Gunung Panti Forest Reserve (GPFR). File: oo_910223.xlsx https://binary.pensoft.net/file/910223 Farah Farhana Ramli

## Figures and Tables

**Table 1.** The checklist of herpetofauna species in Gunung Belumut Amenity Forest, Johor.

**Family**	**Species**	**No. of Individuals**	**Relative Abundance (%)**	**IUCN status**
**Amphibia**				
Bufonidae	*Phrynoidisasper*	12	7.0	Least Concern
Dicroglossidae	*Fejervaryalimnocharis*	4	2.3	Least Concern
	*Limnonectesblythii*	59	34.3	Least Concern
	*Limnonectesdeinodon*	21	12.2	Least Concern
	*Limnonectesplicatellus*	3	1.7	Least Concern
	*Occidozygamartensii*	2	1.2	Least Concern
Microhylidae	*Kalophrynuslimbooliati*	4	2.3	Least Concern
	*Microhylabutleri*	1	0.6	Least Concern
	*Microhylamukhlesuri*	7	4.1	Least Concern
Ranidae	*Hylaranalabialis*	26	15.1	Least Concern
	*Hylaranamiopus*	3	1.7	Least Concern
	*Hylaranasundabarat*	1	0.6	Least Concern
	*Hylaranabaramica*	1	0.6	Least Concern
	*Odorranahosii*	18	10.5	Least Concern
Rhacophoridae	*Polypedatesdiscantus*	2	1.2	Not Available
	*Polypedatesleucomystax*	6	3.5	Least Concern
	*Polypedatesmacrotis*	1	0.6	Least Concern
	*Rhacophorusnorhayatiae*	1	0.6	Least Concern
**Reptiles**				
Agamidae	*Dracomelanopogon*	2	1.0	Least Concern
	*Dracofimbriatus*	1	0.5	Least Concern
	*Gonocephalusgrandis*	5	2.4	Least Concern
	*Aphaniotisfusca*	2	1.0	Least Concern
Colubridae	*Gonyosomaoxycephalum*	1	0.5	Least Concern
	*Dendrelaphiscyanochloris*	1	0.5	Least Concern
	*Ptyasfusca*	1	0.5	Least Concern
Elapidae	*Calliophisintestinalis*	1	0.5	Least Concern
Gekkonidae	*Cyrtodactylusconsobrinus*	8	3.8	Least Concern
	*Cyrtodactylussworderi*	1	0.5	Endangered
	*Hemidactyluscraspedotus*	2	1.0	Least Concern
	*Gekkohulk*	1	0.5	Least Concern
Pythonidae	*Malayopythonreticulatus*	1	0.5	Least Concern
Scincidae	*Eutropismacularia*	1	0.5	Least Concern
	*Eutropismultifasciata*	5	2.4	Least Concern
	*Subdolusepsbowringii*	1	0.5	Least Concern
Trionychidae	*Doganiasubplana*	1	0.5	Least Concern
Varanidae	*Varanussalvator*	1	0.5	Least Concern
Viperidae	*Trimeresurushageni*	1	0.5	Least Concern
	*Tropidolaemuswagleri*	1	0.5	Least Concern

**Table 2.** The Diversity indices of amphibians and reptiles in Gunung Belumut Amenity Forest, Johor.

**Diversity Index**	**Amphibians**	**Reptiles**
Species richness (S)	18	20
Dominance (D)	0.18	0.10
Shannon (H')	2.15	2.67
Evenness (e^H/S)	0.48	0.72
Chao 1 Estimator	21.33	42.75
